# Supplementary material for: Murine Long Noncoding RNA Morrbid Contributes in the Regulation of NRAS Splicing in Hepatocytes In Vitro
Source: Int J Mol Sci. 2020 Aug 5;21(16):5605. doi: 10.3390/ijms21165605 (PMC7460575; doi:10.3390/ijms21165605)
Supplement: Supplementary file 1 [file ijms-21-05605-s001.zip › Suppl F 3/NRAS Supplementary_v7.docx]

Murine Long Noncoding RNA Morrbid Contributes in the Regulation of NRAS Splicing in Hepatocytes In Vitro

Anna Fefilova ^1^, Pavel Melnikov ^2^, Tatiana Prikazchikova ^1^, Tatiana Abakumova ^1^, Ilya Kurochkin ^1^, Pavel V. Mazin ^1^, Rustam Ziganshin ^3^, Olga Sergeeva ^1,^* and Timofei S. Zatsepin ^1,4,^*

^1^ Center of Life Sciences, Skolkovo Institute of Science and Technology, 121205 Moscow, Russia.; Anna.Fefilova@skolkovotech.ru (A.F.); T.Prikazchikova@skoltech.ru (T.P.); T.Abakumova@skoltech.ru (T.A.); Ilia.Kurochkin@skolkovotech.ru (I.K.); p.mazin@skoltech.ru (P.V.M.)

^2^ Serbsky National Medical Research Center for Psychiatry and Narcology, 119034 Moscow, Russia; proximopm@gmail.com

^3^ Shemyakin-Ovchinnikov Institute of Bioorganic Chemistry, 117997 Moscow, Russia; Rustam.Ziganshin@gmail.com

^4^ Department of Chemistry, Lomonosov Moscow State University, 119992 Moscow, Russia.

***** Correspondence: O.Sergeeva@skoltech.ru (O.S.), T.Zatsepin@skoltech.ru (T.S.Z.)

**Supplementary**

**Supplementary Table S1**. List of ASOs and siRNAs used in the study.

| **Name** | **Sequence** |
| --- | --- |
| Morrbid ASOs | |
| Morrbid ASO-1 | usgsuscscsAsCsAsTsGsAsTsTsAsGsgsasasasas |
| Morrbid ASO-2 | gsuscscscsCsTsCsAsTsTsCsTsCsAsgsasgsasus |
| Morrbid ASO-3 | ususgscsusTsTsTsTsAsAsTsGsAsAsgsasasasgs |
| Morrbid ASO-4 | csusgsusgsasAsGsAsTsCsCsCsAsAsGsasusasusgscs |
| Morrbid ASO-5 | gsasusasgsAsCsGsGsGsTsCsCsGsCscsusgscscs |
| Morrbid ASO-6 | asgsasgscsAsTsCsCsGsAsAsAsAsGscsuscsusgs |
| Morrbid ASO-7 | cscscsasgscsAsCsCsCsGsTsGsAsGsCsascsusgsasgs |
| Morrbid ASO-8 | cscsgsususCsCsCsGsGsGsTsGsGsGsasascscscs |
| Morrbid ASO-9 | gscsasgscsAsGsGsTsAsGsGsGsGsTsgscsuscscs |
| Morrbid ASO-10 | ascsgsuscsCsTsGsAsTsTsTsTsTsCsusgsusgsus |
| Morrbid ASO-11 | gsasusasasCsAsAsAsCsCsAsGsCsTsgsusasasgs |
| Morrbid ASO-12 | gscscsasasusAsAsAsTsAsAsAsGsTsAsasusasgsasus |
| Morrbid ASO-13 | csuscscscsAsGsCsCsGsTsGsAsTsCsasgscsasgs |
| Upper case – 2’-deoxynucleotide, lower case – 2’-O-methylribonucleotide, s –phosphorothioate group. | |
| SFPQ siRNAs | |
| msSfpQ-1 | GuAuGAAGGGccAAAuAAATsT |
|  | UUuAUUUGGCCCUUCAUACTsT |
| msSfpQ-2 | cAuuAAGcuuGAAucuAGATsT |
|  | UCuAGAUUcAAGCUuAAUGTsT |
| msSfpQ-3 | ccAGAAGAAuccAAuGuAuTsT |
|  | AuAcAUUGGAUUCUUCUGGTsT |
| msSfpQ-4 | AAAcAuGAAGGAuGcuAAATsT |
|  | UUuAGcAUCCUUcAUGUUUTsT |
| msSfpQ-5 | uAuuGAAAGGGcuuGuuGuATsT |
|  | uAcAAcAGCCCUUUcAAuATsT |
| msSfpQ-6 | cuGuCuGuucGAAAucucuTsT |
|  | AGAGAUUUCGAAcAGAcAGTsT |
| control | cuuAcGcuGAGuAcuucGATsT |
|  | UCGAAGuACUcAGCGuAAGTsT |
| UPF siRNA | |
| UPF-1 siRNA | cuGcGuGGuuuAcuGuAAuTsT |
|  | uAUGUUCUGGuACUGGuAGTsT |

Upper case – ribonucleotide, lower case – 2’-O-methylribonucleotide, s –phosphorothioate group.

**Supplementary Table S3**. Differential alternative splicing genes in response to Morrbid depletion.

| **AS Type** | **Gene name** | **Gene ID** |
| --- | --- | --- |
| Alternative Acceptor | Chd4 | ENSMUSG00000063870 |
| Alternative Acceptor | Atp2c1 | ENSMUSG00000032570 |
| Alternative Acceptor | Myrf | ENSMUSG00000036098 |
| Alternative Acceptor | Brd2 | ENSMUSG00000024335 |
| Alternative Acceptor | Map4k4 | ENSMUSG00000026074 |
| Alternative Acceptor | Sbds | ENSMUSG00000025337 |
| Alternative Acceptor | Hax1 | ENSMUSG00000027944 |
| Alternative Acceptor | Atxn2l | ENSMUSG00000032637 |
| Alternative Acceptor | Hnrnpa2b1 | ENSMUSG00000004980 |
| Alternative Acceptor | Oaz1 | ENSMUSG00000035242 |
| Alternative Acceptor | Rpl3 | ENSMUSG00000060036 |
| Alternative Acceptor | Psmd2 | ENSMUSG00000006998 |
| Alternative Acceptor | Msln | ENSMUSG00000063011 |
| Alternative Acceptor | Use1 | ENSMUSG00000002395 |
| Alternative Acceptor | 1600012H06Rik | ENSMUSG00000050088 |
| Alternative Donor | Rbm25 | ENSMUSG00000010608 |
| Alternative Donor | Smarcad1 | ENSMUSG00000029920 |
| Alternative Donor | Herc2 | ENSMUSG00000030451 |
| Alternative Donor | Taf1d | ENSMUSG00000031939 |
| Alternative Donor | Gnl3 | ENSMUSG00000042354 |
| Alternative Donor | Gapdh | ENSMUSG00000057666 |
| Alternative Donor | Akt1s1 | ENSMUSG00000011096 |
| Alternative Donor | Rnasek | ENSMUSG00000093989 |
| Alternative Donor | Gapdh | ENSMUSG00000057666 |
| Alternative Donor | Zfp574 | ENSMUSG00000045252 |
| Alternative Donor | Calu | ENSMUSG00000029767 |
| Alternative Donor | 2610507B11Rik | ENSMUSG00000010277 |
| Alternative Donor | Spg20 | ENSMUSG00000036580 |
| Alternative Donor | Chmp2a | ENSMUSG00000033916 |
| Alternative Donor | Gapdh | ENSMUSG00000057666 |
| Alternative Donor | Ei24 | ENSMUSG00000062762 |
| Alternative Donor | Dnaja1 | ENSMUSG00000028410 |
| Alternative Donor | Tomm34 | ENSMUSG00000018322 |
| Alternative Donor | Spg20 | ENSMUSG00000036580 |
| Alternative Donor | Dcaf8 | ENSMUSG00000026554 |
| Alternative Donor | Emc6 | ENSMUSG00000047260 |
| Alternative Donor | Mboat7 | ENSMUSG00000035596 |
| Alternative Donor | Ptprn | ENSMUSG00000026204 |
| Alternative Donor | Zkscan17 | ENSMUSG00000020472 |
| Alternative Donor | Sf1 | ENSMUSG00000024949 |
| Alternative Donor | Cog8 | ENSMUSG00000031916 |
| Alternative Donor | Cited2 | ENSMUSG00000039910 |
| Alternative Donor | Cyc1 | ENSMUSG00000022551 |
| Alternative Donor | Snhg1 | ENSMUSG00000108414 |
| Alternative Donor | Trim3 | ENSMUSG00000036989 |
| Alternative Donor | Hhex | ENSMUSG00000024986 |
| Alternative Donor | Srsf5 | ENSMUSG00000021134 |
| Alternative Donor | Nin | ENSMUSG00000021068 |
| Alternative Donor | Dnaja1 | ENSMUSG00000028410 |
| Alternative Donor | Pafah1b2 | ENSMUSG00000003131 |
| Alternative Donor | Rack1 | ENSMUSG00000020372 |
| Alternative Donor | Pttg1 | ENSMUSG00000020415 |
| Alternative Donor | Stk16 | ENSMUSG00000026201 |
| Cassette Exon | Commd1 | ENSMUSG00000051355 |
| Cassette Exon | Sh3pxd2a | ENSMUSG00000053617 |
| Cassette Exon | Kmt5a | ENSMUSG00000049327 |
| Cassette Exon | Tra2b | ENSMUSG00000022858 |
| Cassette Exon | Mfge8 | ENSMUSG00000030605 |
| Cassette Exon | Tpm1 | ENSMUSG00000032366 |
| Cassette Exon | Gnb1 | ENSMUSG00000029064 |
| Cassette Exon | Ptpn12 | ENSMUSG00000028771 |
| Cassette Exon | Bub3 | ENSMUSG00000066979 |
| Cassette Exon | Dhx9 | ENSMUSG00000042699 |
| Cassette Exon | Ube2i | ENSMUSG00000015120 |
| Cassette Exon | Vkorc1l1 | ENSMUSG00000066735 |
| Cassette Exon | Chmp2a | ENSMUSG00000033916 |
| Cassette Exon | Fam20b | ENSMUSG00000033557 |
| Cassette Exon | Nras | ENSMUSG00000027852 |
| Cassette Exon | Cystm1 | ENSMUSG00000046727 |
| Cassette Exon | Srsf3 | ENSMUSG00000071172 |
| Cassette Exon | Xiap | ENSMUSG00000025860 |
| Cassette Exon | Arl6ip1 | ENSMUSG00000030654 |
| Cassette Exon | Actn1 | ENSMUSG00000015143 |
| Cassette Exon | Slc25a39 | ENSMUSG00000018677 |
| Cassette Exon | Hdgf | ENSMUSG00000004897 |
| Cassette Exon | Impdh2 | ENSMUSG00000062867 |
| Cassette Exon | Actr3 | ENSMUSG00000026341 |
| Cassette Exon | Snrpb | ENSMUSG00000027404 |
| Cassette Exon | Epn1 | ENSMUSG00000035203 |
| Cassette Exon | Srrm2 | ENSMUSG00000039218 |
| Cassette Exon | Furin | ENSMUSG00000030530 |
| Cassette Exon | Ndufa6 | ENSMUSG00000022450 |
| Cassette Exon | Pkm | ENSMUSG00000032294 |
| Retained Intron | Aurkaip1 | ENSMUSG00000065990 |

**Supplementary Table S4**. List of PCR primers used in the study.

| **Name** | **Sequence, 5’→3’** |
| --- | --- |
| GAPDH_FWD | TGCACCACCAACTGCTTAGC |
| GAPDH_REV | GGATGCAGGGATGATG |
| Morrib_FWD | AAATGACACAGACACAGAAAAATCA |
| Morrbid_REV | ACTGAGTAGCTAAGAGTCCGTTCC |
| PTC junction_FWD | CCCACCATAGAGGATTCTTACC |
| PTC junction_REV | GATCCCACCATAGAGGATTCTTACCG |
| PTC down_FWD | CAGATATAAATTCACCTGCCCTTATGT |
| PTC down_REV | GTAGAGGTTAATATCTGCAAATGATTTG |
| NRAS no PTC_FWD | GGTCTCACTGCACTACCCTG |
| NRAS no PTC_REV | ATAATCACACGCATGCATGCAC |
| NRAS total_FWD | ACGAACTGGCCAAGAGTTACG |
| NRAS total_REV | CATTCGGTACTGGCGTATCTCC |
| NRAS total 2_FWD | ACATGAGGACAGGCGAAGG |
| NRAS total 2_REV | AGCACCATGGGGACATCATC |
| m_SFPQ_FWD | GGAAGCGACATGCGTACTGA |
| m_SFPQ_REV | TTCCAGGCCCCATTCCTCTA |
| UPF1_FWD | AGATCACGGCACAGCAGAT |
| UPF1_REV | TGGCAGAAGGGTTTTCCTT |
| snU6_FWD | CGCTTCGGCAGCACATATAC |
| snU6_REV | AAATATGGAACGCTTCACGA |
| Nras_T7_PCR_fwd1 | TAATACGACTCACTATAGGGAAAAGCGCCTTGACGATCC |
| Nras_noT7_PCR_rev1 | GCAAATACACAGAGGAACCCTTCGCC |
| Nras_noT7_PCR_fwd1 | GGAAAAGCGCCTTGACGATCC |
| Nras_T7_PCR_rev1 | TAATACGACTCACTATAGGCAAATACACAGAGGAACCCTTCGCC |
| NRAS_ex1-intr1_fwd | CCACTTTGTGGATGAATATGATCCCAC |
| NRAS_ex1-intr1_rev | CCTCCTTGCTTTCTCTTCTCTTTACT |

**Supplementary Table S5**. Biotinylated Probes used in CHART and RIP protocols.

| **Name** | **Sequence** |
| --- | --- |
| Morrbid_biot_1 | Biotin-s-csusgsusgsasAsGsAsTsCsCsCsAsAsGsasusasusgsc-s-NH2 |
| Morrbid_biot_2 | Biotin-s-gsasusasgsAsCsGsGsGsTsCsCsGsCscsusgscsc-s-NH2 |
| Morrbid_biot_3 | Biotin-s- cscscsasgscsAsCsCsCsGsTsGsAsGsCsascsusgsasg-s-NH2 |
| Morrbid_biot_4 | Biotin-s- csuscscscsAsGsCsCsGsTsGsAsTsCsasgscsasg-s-NH2 |
| Control_1 | Biotin-s-csasgsasgsasgscsTsCsAsCsAsCsTsTsCsAsasasasusgsuscsc-s-NH2 |
| Control_2 | Biotin-s-csusgsasgsasgsusAsGsGsTsTsTsGsTsTsTscscsasgsgsasa-s-NH2 |
| Control_3 | Biotin-s-gsgsususasusgsusTsCsCsTsAsGsTsGsAsCsAsgsasasasgsasgsu-s-NH2 |
| Control_4 | Biotin-s-gscsasususcscscsAsTsCsAsCsAsTsCsTsCsTscsusasgsusgsusg-s-NH2 |

Upper case – 2’-deoxynucleotide, lower case – 2’-O-methylribonucleotide, s –phosphorothioate group, NH2 – 3’-hexylamine.


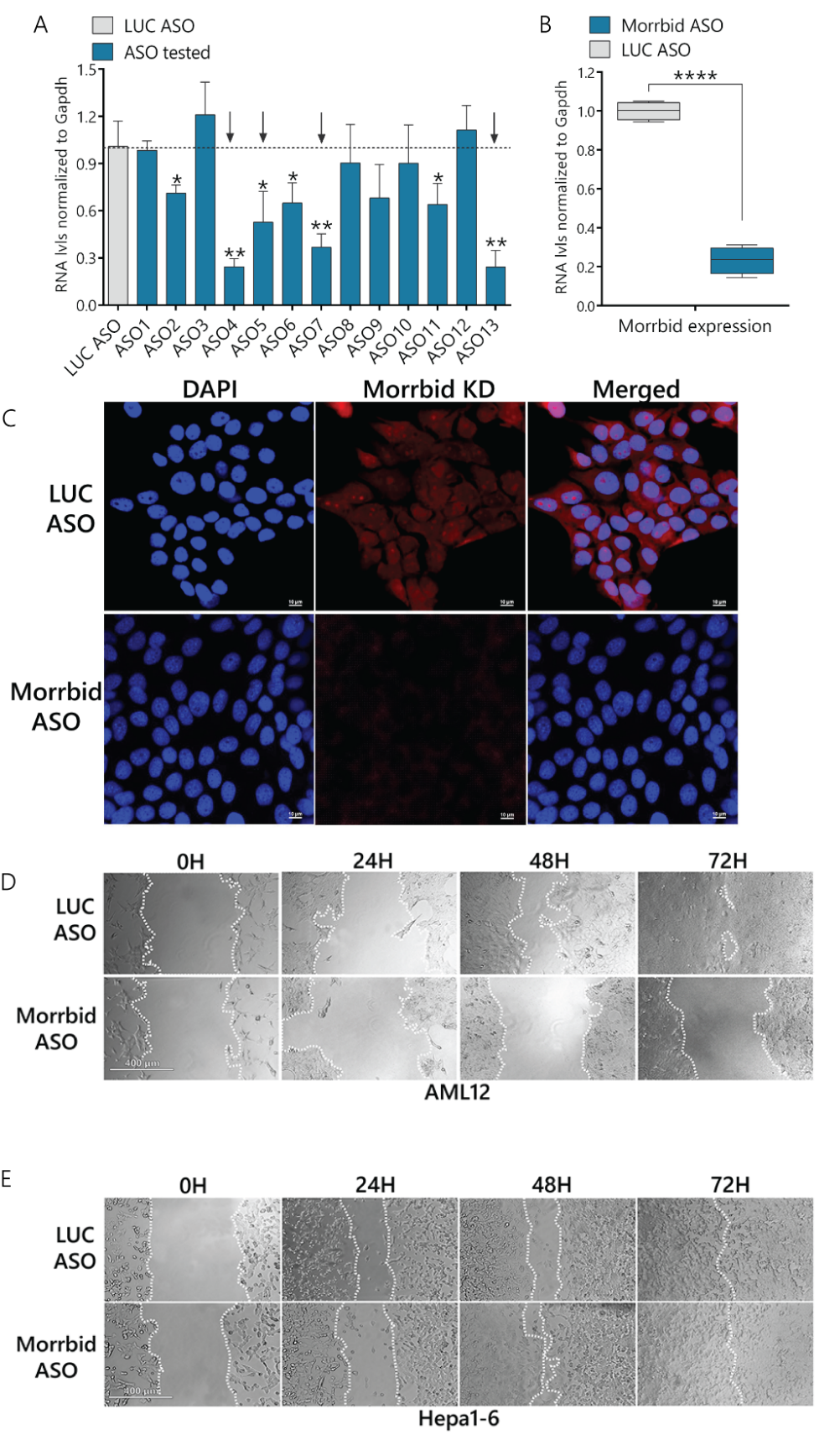


**Figure S1**. **(A)**Estimation the efficacy of ASOs targeting Morrbid in AML12 cells after 24 h by RT-qPCR. **(B)** Efficacy of Morrbid lncRNA inhibition using the mix of 4 most efficient ASOs (ASO4, ASO5, ASO7, ASO13) analysed by RT-qPCR. **(C)** Fluorescent in situ hybridization analysis of Morrbid depletion in AML12 cells after 24h of inhibition by ASOs. DNA was stained with Dapi, Morrbid was stained with Cy5-labeled probes. **(D)** Microscopy images of wound healing assay in AML12 control and Morrbid KD cells **(E)** Microscopy images of wound healing assay in Hepa1-6 control and Morrbid KD cells. Results show mean ± SD, * p < 0.05, ** p < 0.01 and **** p < 0.0001.


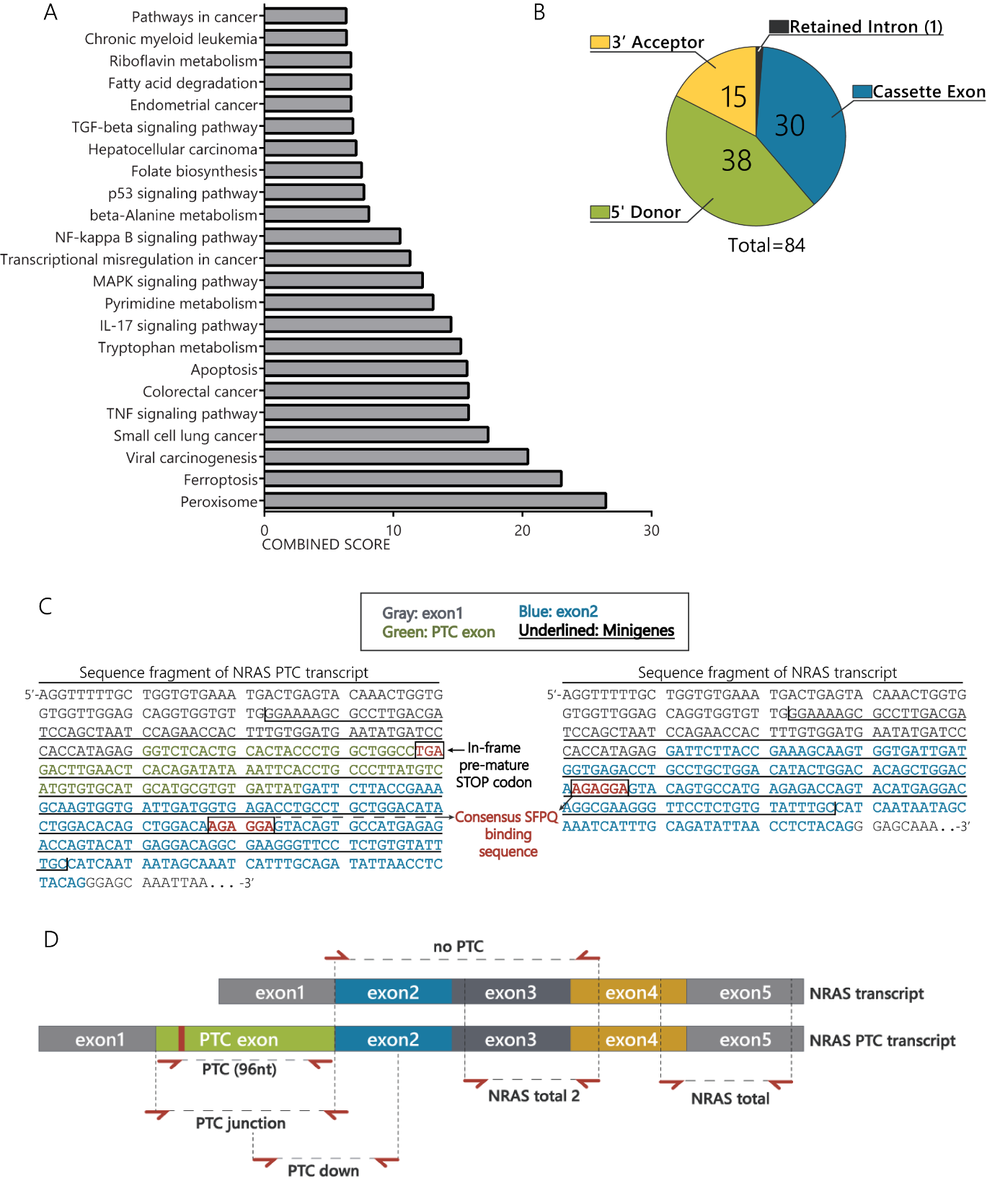


**Figure S2**. **(A)** KEGG enrichment analysis of genes differentially expressed in AML12 cells after 2 days of Morrbid knockdown. **(B)** Summary of differential changes in alternative splicing in Morrbid KD cells. **(C)** Fragments of NRAS PTC and NRAS no PTC transcripts with designated exon1, PTC exon, exon2, positions of pre-mature STOP-codon, consensus SFPQ binding sites, generated NRAS minigenes. **(D)** Schematic representation of qPCR primers positions used in the study to detect various isoforms of NRAS.


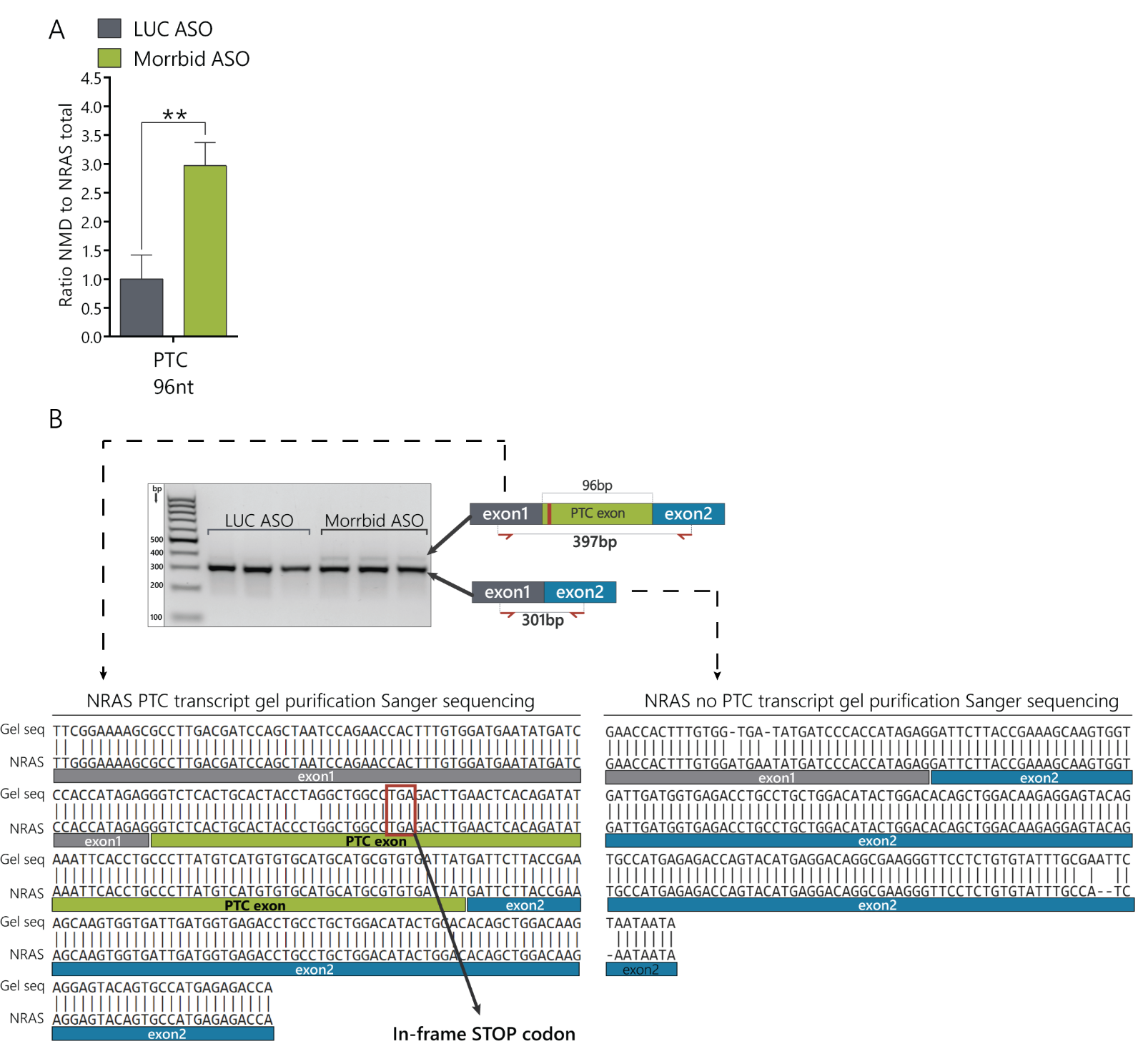


**Figure S3.** **(A)** RT-qPCR analysis of relative NRAS PTC transcript expression in control and Morrbid KD cells using primers PTC (96nt). **(B)** Alignment the Sanger sequence data of NRAS isoforms PCR products spanning across NRAS cassette exon on the NRAS genome data (NCBI Gene ID: 18176). Results show mean ± SD ** p < 0.01.


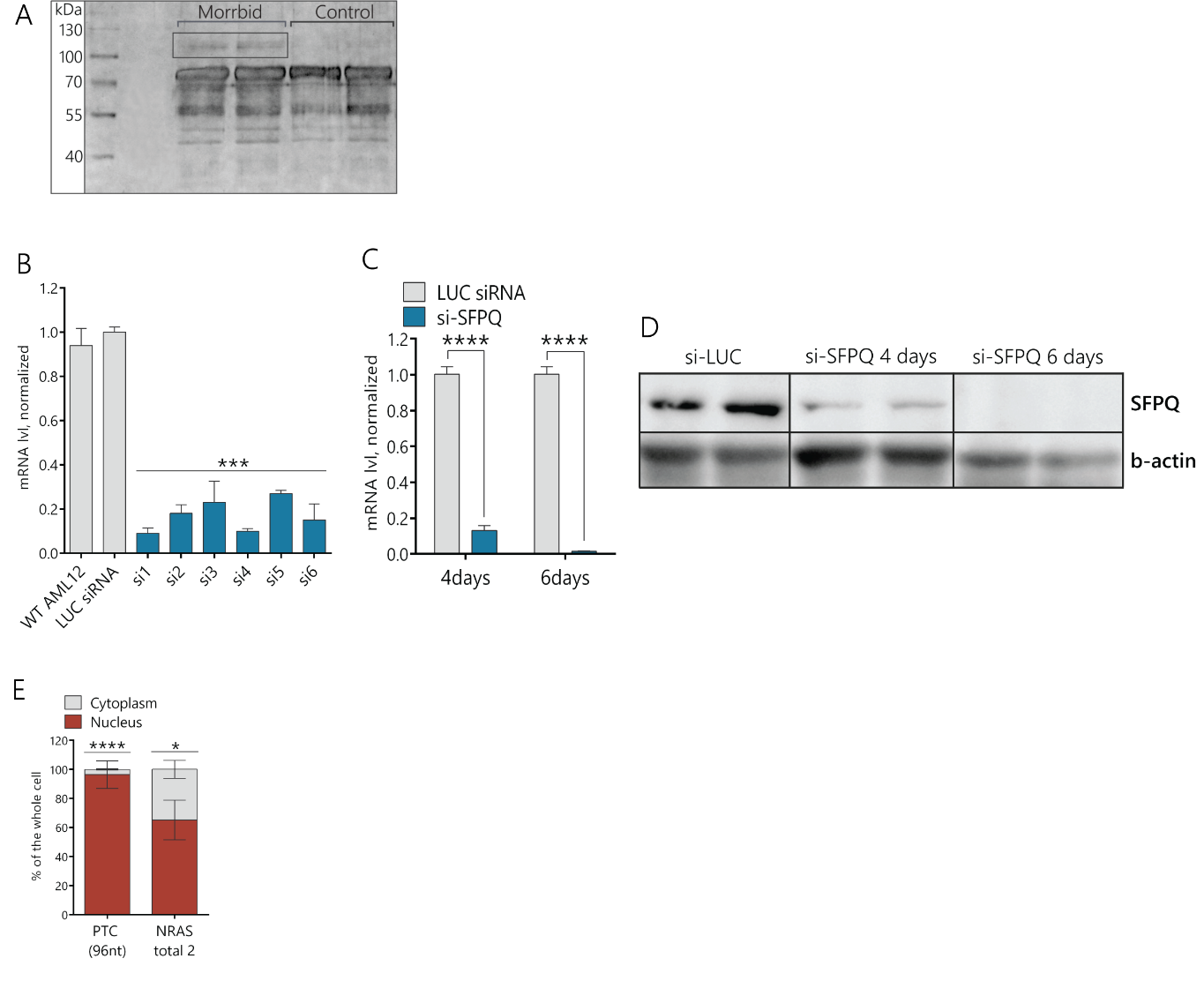


**Figure S4**. **(A)** SDS-PAGE of samples obtained in CHART experiment**,** framed are Morrbid specific bands, excised and analyzed by LC-MS. **(B)** Efficiency test of siRNAs targeting SFPQ mRNA in AML12 cells after 24h of KD analyzed by RT-qPCR. **(C-D)** Estimation SFPQ mRNA **(C)** and protein **(D)** level after RNAi-mediated inhibition by RT-qPCR and Western-blot respectively. **(E)** RT-qPCR analysis of NRAS total and NRAS PTC transcripts in the nuclear and cytoplasmic fractions extracted from AML12 cells. Results show mean ± SD, * p < 0.05, *** p < 0.001, **** p<0.0001.
